# Increased HEV Seroprevalence in Patients with Autoimmune Hepatitis

**DOI:** 10.1371/journal.pone.0085330

**Published:** 2014-01-21

**Authors:** Sven Pischke, Anett Gisa, Pothakamuri Venkata Suneetha, Steffen Björn Wiegand, Richard Taubert, Jerome Schlue, Karsten Wursthorn, Heike Bantel, Regina Raupach, Birgit Bremer, Behrend Johann Zacher, Reinhold Ernst Schmidt, Michael Peter Manns, Kinan Rifai, Torsten Witte, Heiner Wedemeyer

**Affiliations:** 1 Department of Gastroenterology, Hepatology and Endocrinology, Hannover Medical School, Hannover, Germany; 2 Institute for Pathology, Hannover Medical School, Hannover, Germany; 3 Department of Clinical Immunology, Hannover Medical School, Hannover, Germany; University of South Carolina School of Medicine, United States of America

## Abstract

**Background:**

Hepatitis E virus (HEV) infection takes a clinically silent, self-limited course in the far majority of cases. Chronic hepatitis E has been reported in some cohorts of immunocompromised individuals. The role of HEV infections in patients with autoimmune hepatitis (AIH) is unknown.

**Methods:**

969 individuals were tested for anti-HEV antibodies (MP-diagnostics) including 208 patients with AIH, 537 healthy controls, 114 patients with another autoimmune disease, rheumatoid arthritis (RA), and 109 patients with chronic HCV- or HBV-infection (HBV/HCV). Patients with AIH, RA and HBV/HCV were tested for HEV RNA. HEV-specific proliferative T cell responses were investigated using CFSE staining and in vitro stimulation of PBMC with overlapping HEV peptides.

**Results:**

HEV-antibodies tested more frequently positive in patients with AIH (n = 16; 7.7%) than in healthy controls (n = 11; 2.0%; p = 0.0002), patients with RA (n = 4; 3.5%; p = 0.13) or patients with HBV/HCV infection (n = 2; 2.8%; p = 0.03). HEV-specific T cell responses could be detected in all anti-HEV-positive AIH patients. One AIH patient receiving immunosuppression with cyclosporin and prednisolone and elevated ALT levels had acute hepatitis E but HEV viremia resolved after reducing immunosuppressive medication. None of the RA or HBV/HCV patients tested HEV RNA positive.

**Conclusions:**

Patients with autoimmune hepatitis but not RA or HBV/HCV patients are more likely to test anti-HEV positive. HEV infection should been ruled out before the diagnosis of AIH is made. Testing for HEV RNA is also recommended in AIH patients not responding to immunosuppressive therapy.

## Introduction

Autoimmune hepatitis (AIH) is an immune mediated liver disease more often affecting women than men. AIH is characterised by elevated serum IgG levels, the presence of certain autoantibodies and distinct histological features in the absence of other causes of liver disease [1]. The underlying pathomechanisms leading to autoimmune hepatitis are not well defined. One possibility is that viral infections trigger break of immunotolerance. Already 20 years ago an association between herpes simplex virus 1 (HSV 1) infection and autoimmune hepatitis has been described [2], [3]. Other infectious agents including hepatitis C virus (HCV), cytomegalovirus, human T lymphotropic viruses 1 and 2 or salmonella typhimurum have been suggested to induce autoimmune liver disease [4]. If infections with the hepatitis E virus (HEV) are associated with AIH is unknown.

HEV infection takes a clinically silent course in the far majority of patients [5]. Few subjects may develop acute liver disease which can take a more severe course in particular in pregnant women or patients with underlying chronic liver diseases [6]. In recent years it became evident that HEV infection is not necessarily self limiting in all cases but may progress to chronic infection in immuno-compromised individuals [5], [7]. Chronic hepatitis E has been described in liver and kidney transplant recipients [8] and also in some HIV-infected patients [9]. In Northern Germany, chronic hepatitis E was identified as the cause of graft hepatitis in 3% of liver transplant recipients with elevated liver enzymes [10]. Importantly persistent HEV infections have been associated with progressive liver disease [7], [8], [10], [11]. To what extend HEV infections may lead to chronic hepatitis E in other patient groups receiving immunosuppressive medications including patients with autoimmune liver disease and rheumatoid arthritis is currently unknown.

The aims of this study were therefore, (i) to investigate the prevalence of antibodies to HEV in patients with autoimmune hepatitis and (ii) to determine if AIH patients receiving standard immunosuppressive medications are at risk for chronic hepatitis E in a low endemic Central European country and (iii) to rule out that rare cases of immunocompetent patients with chronic HEV infection had been misdiagnosed as autoimmune hepatitis.

## Methods

From October 2009 until March 2010 all consecutive patients with AIH (n = 127) presenting our outpatient clinic were tested prospectively for presence of HEV RNA and anti HEV IgG. In addition, a control group of patients with viral hepatitis B or C (n = 109) was recruited. The diagnosis of autoimmune hepatitis was based on internationally accepted criteria [12]. Patients after liver transplantation were excluded. To compare the results with a cohort of patients with another autoimmune disease 114 consecutive patients receiving immunosuppressive medications followed by our Rheumatology outpatient clinic were studied between January 2012 and March 2012.

To enlarge the overall study cohort of patients with AIH, 81 additional patients were studied retrospectively. All retrospectively investigated patients were recruited at Hannover Medical School between 1998 and 2008. Furthermore 537 healthy subjects (employees of Hannover Medical School (n = 167) and blood donors (n = 370)) were studied for anti-HEV as already described as part of another project [10].

All AIH, HBV/HCV and RA patients were tested for the presence of HEV IgG antibodies by the MP assay (MP Biomedicals, formerly Genelabs Diagnostics, Singapore) according to the manufacture's instruction. Details of the primers used in the nested HEV RNA PCR were reported previously (11). Healthy subjects were only tested for anti-HEV-IgG.

Most AIH-patients received standard immunosuppressive regiments according to current guidelines [13] including mainly corticosteroids and partially azathioprine. Overall 70% of the AIH patients were female (n = 145). Age, gender and ALT levels of the overall study cohort are shown in table 1.

**Table 1:** Prevalence of HEV antibodies and HEV-RNA in different patient groups.

	Patients with AIH (n = 208)	Patients with HBV or HCV infections (n = 109)	Patients with RA (n = 114)
Male	63 (30%)	59 (54%)**	29 (25%)
Age in years, mean (range, SD)	51 (18–84, 15)	49 (18–79, 13)	59 (19–85, 14)**
AST in U/ml	71 (15–684, 103)	69 (15–369, 59%)*	26 (13–63, 9)**
ALT in U/ml	94 (8–1422, 181)	82 (12–419, 78), p = 0.003**	26 (8–340, 32)**
HEV RNA positive	1 (0.5%)	0 (0%)	0 (0%)
Anti HEV IgG positive	16 (7.7%)	2 (1.8%)*	4 (3.5%)

p-values for comparison with AIH patients:

* p<0.05;

** p-values<0.01.

A subset of AIH patients (n = 123) was tested in addition with the Wantai anti-HEV assay (Wantai, Bejing). The frequency of anti-HEV in this subset was compared to 90 additional immunocompetent patients with chronic hepatitis B or C. Borderline test results were considered as positive for further statistical analysis.

### Analysis of HEV-specific T cell responses

Cellular immune responses against HEV were studied in 1 AIH-patient with acute hepatitis E who tested HEV RNA and anti-HEV positive. Additional samples could be investigated from this subject 2 years after recovery. Furthermore, 4 anti-HEV-positive patients were tested for HEV-specific T cell responses. PBMC were stimulated with HEV overlapping peptide pools (spanning ORF2 and ORF3) as described previously [14], [15]. T-cell proliferation was measured after 7 days by CFSE (5, 6-carboxyfluorescein diacetate succinimidyl ester) assay [14], [15].

### Statistics

Data for the different patient groups are presented as means and standard deviations. A comparison of continuous and categorical data between groups was performed using the chi-square test. Comparison of quantitative data between groups was performed using the Mann-Whitney test. A *p-*value<0.05 was considered significant.

### Ethics

This study (including the analysis of HEV specific T-cell responses) was approved by the local research Ethics Committee (Ethics Committee of Hannover Medical School, Hannover, Germany). No research was conducted outside of our country. Patients agreed to hepatitis E testing as part of routine clinical work-up and thus no written informed consent was required. Retrospective testing of 81 samples from AIH patients was performed as part of the approved protocol and data were analysed anonymously according to the institutional and national ethics rules.

The need for written informed consent of participants of this study has been waived by the institutional review board according to our guidelines.

## Results

### Prevalence of HEV antibodies in patients with autoimmune hepatitis or persistent HBV- or HCV-infections

**Figure 1:**
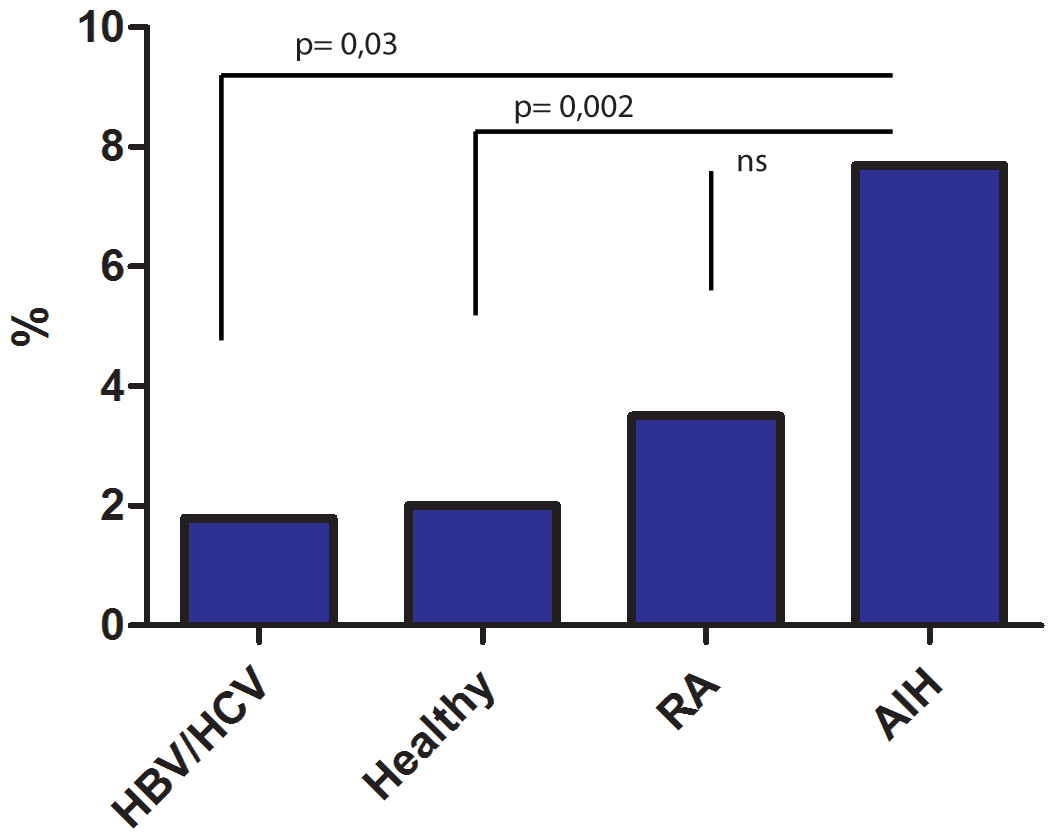
Seroprevalence rate of HEV specific antibodies in healthy controls, patients with chronic HBV- or HCV-infection and patients with autoimmune hepatitis.

Anti HEV antibodies were detected in 16 out of 208 patients with autoimmune hepatitis (7.7%; 95% confidence interval 4.8–12). In contrast, anti HEV antibodies were detected in only 2/109 of control subjects with HCV or HBV infection (1.8%; CI 0.6–6.4). RA patients and healthy controls tested anti-HEV positive in 3.5% (4/115; CI 1.4–8.6) and 2.0% (11/537; CI 1.2–3.6), respectively (figure 1). Thus, anti HEV antibodies were more frequent in AIH patients than subjects with chronic viral hepatitis B or C (p = 0.03) or healthy individuals (p<0.001). The difference between the anti-HEV seroprevalence rate in AIH and RA patients did not reach the level of significance (p = 0.13), however, AIH patients were younger than individuals with RA (mean age 51 years vs. 59 years, Mann-Whitney test: p<0.001).

Anti HEV IgG-positive AIH patients were older than in anti-HEV-negative patients (mean 58 years, SD 13 years vs mean 50 years, SD 15 years, p = 0.032 Mann-Whitney test). There was no difference regarding gender distribution between anti-HEV positive versus negative AIH patients (4/16 male vs 59/192 male, p = 0.44 chi-square test), ALT levels (mean 154 U/ml, SD 347 U/ml vs mean 89 U/ml, SD 160 U/ml, p = 0.26 Mann-Whitney test) or AST levels (mean 95 U/ml, SD 157 U/ml vs mean 69 U/ml, SD 98 U/ml, p = 0.07 Mann-Whitney test).

As this study was based on the anti-HEV MP assay, we next aimed to confirm the findings by a second, independent sero-assay, the Wantai assay, which has been suggested to be more sensitive than the MP assay [16], [17]. Sera of 123 AIH patients and 90 patients with chronic hepatitis B or C were investigated. Indeed, the testing with the Wantai assay confirmed that AIH patients were numerically more likely to test anti HEV positive (33%, CI 25–42) than in HBV/HCV patients (21%, CI 14–31) even though only borderline statistical significance was reached (p = 0.05).

### Prevalence of HEV RNA in patients with autoimmune hepatitis

**Figure 2:**
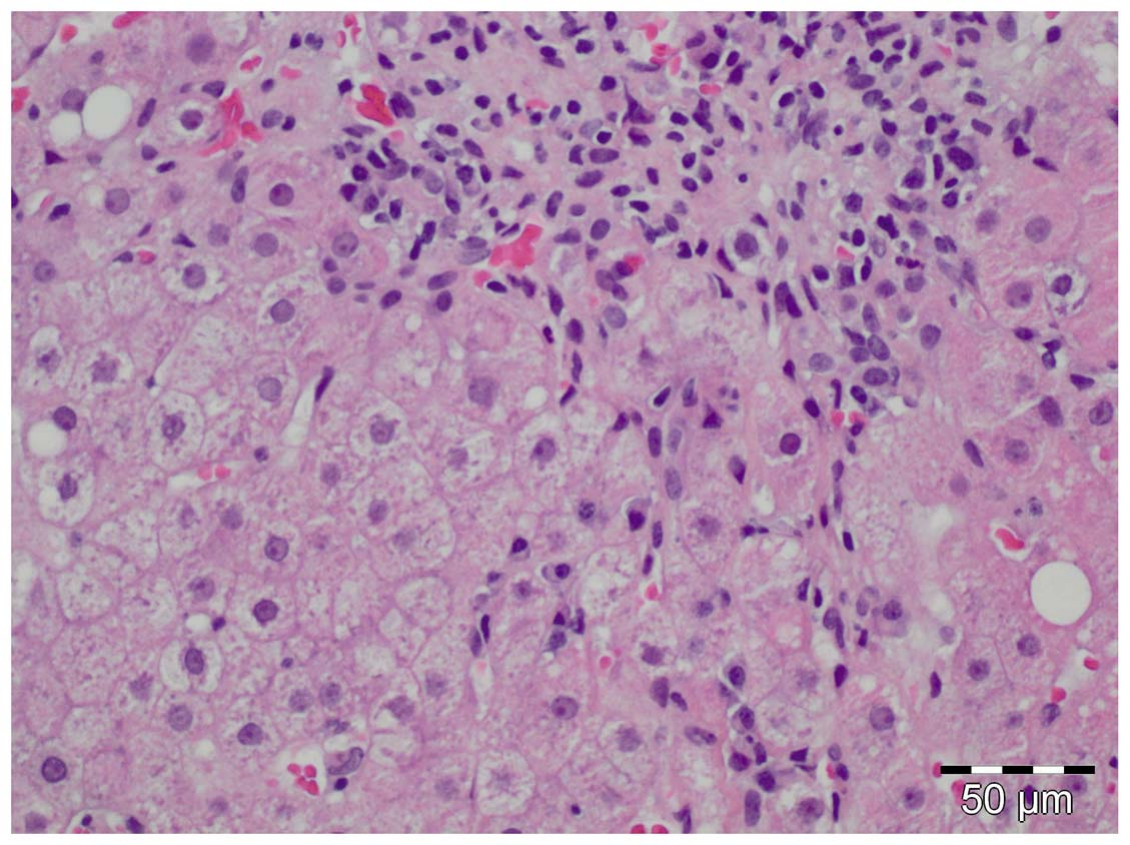
Histology of an autoimmune hepatitis patient with acute HEV infection. (H.E. stain, original magnification 200×).

All patients with AIH, RA or HBV/HCV were also tested for HEV RNA irrespective of the presence of anti HEV antibodies. None of the RA patients or HCV/HBV-infected subjects tested HEV RNA positive. HEV RNA was detected in only one AIH patient. This was a 70 years old female patient in whom AIH had been diagnosed 5 years earlier. Immunosuppressive medication consisted of cyclosporine (125 mg/d) and prednisolone (50 mg/d). The high dose of 50 mg prednisolone had been prescribed as liver enzymes had increased despite treatment with 20 mg prednisolone per day. A liver biopsy was performed showing a lymphoplasmacytic infiltration and signs of acute inflammation and portal fibrosis (Ishak-score A3,B0,C2,D3,F3 and steatosis S1, figure 2). Immunosuppression was reduced after the HEV RNA tested positive. HEV RNA became negative after reducing the dose of prednisolone from 50 mg/d to 15 mg/d. Anti HEV IgG antibodies remained positive. Retrospective analysis of stored serum samples revealed that this patient had no detectable HEV RNA or anti HEV IgG antibodies 4 months before this episode of ALT elevation indicating that the patient had indeed acute but not chronic hepatitis E at the time of investigation.

### HEV-specific T-cell responses

**Figure 3:**
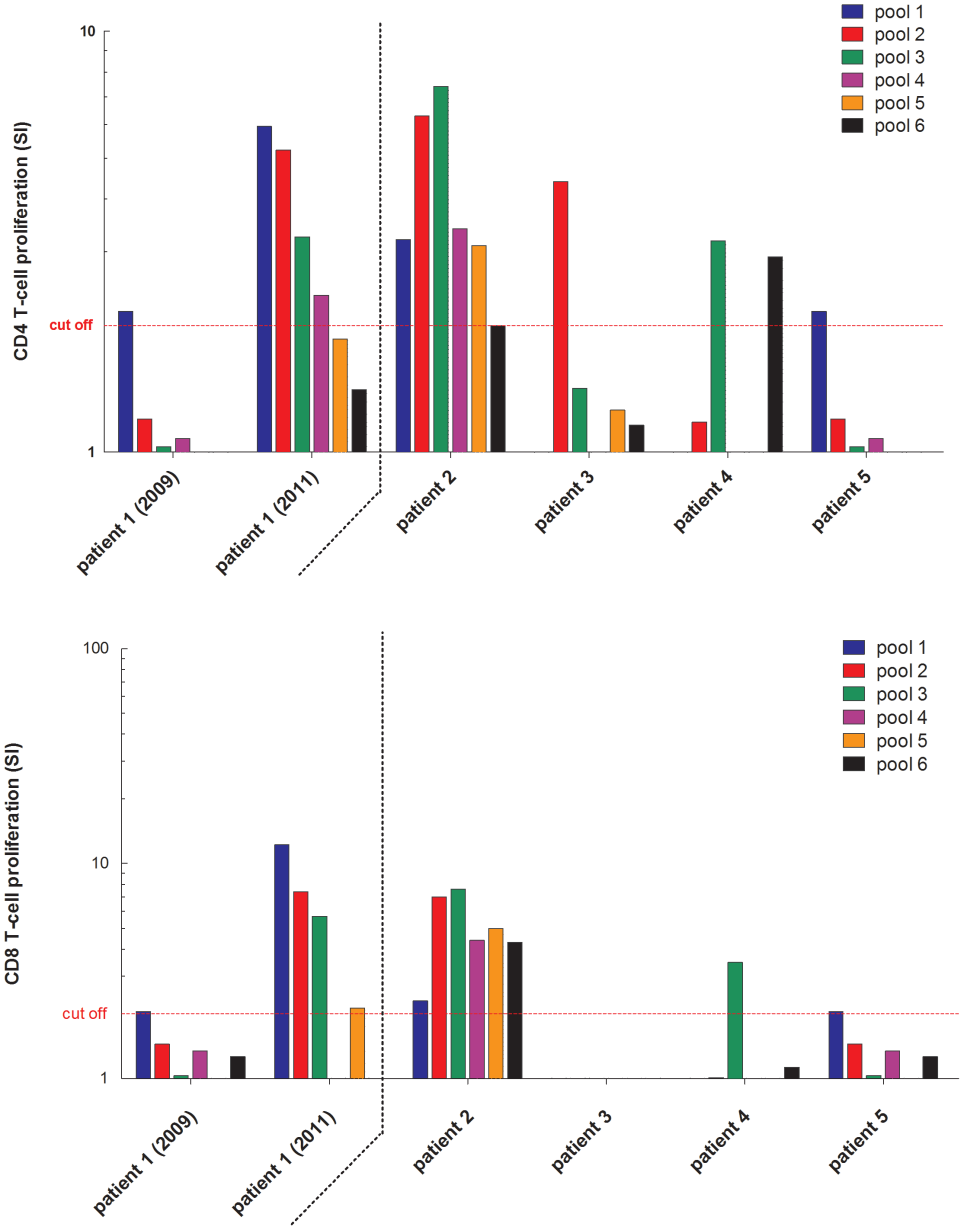
HEV specific T-cell responses in AIH patients (anti-HEV IgG positive).

HEV-specific T cell responses could be studied in the anti-HEV-positive AIH patient, who tested positive for RNA and in 4 further anti-HEV positive AIH patients (figure 3). HEV-specific proliferation of CD4+ T cells was detectable in all 4 anti-HEV-positive/HEV RNA-negative AIH patients with previous HEV infection. In addition, HEV-specific CD8+ T cell responses were detectable in two of the four patients. The AIH-patient with acute hepatitis E showed a low HEV-specific T cell response in the peripheral blood when she was still viremic. However, two years after recovery, a multispecific CD4+ and CD8+ T cell response became detectable (figure 3).

## Discussion

We here show that HEV antibodies may be more frequent in patients with autoimmune hepatitis than in individuals with other chronic viral liver diseases or patients with other autoimmune diseases and healthy controls. We also show that chronic hepatitis E seems not to be a major clinical problem in individuals with autoimmune hepatitis receiving standard immunosuppressive medications. However in single cases HEV infection can occur and may contribute to the severity of hepatitis.

A higher prevalence of anti HEV antibodies in patients with AIH patients has already been described previously in two smaller studies from California [18] and Sweden [19] performed in the 1990ies. Thus, our findings obtained in a larger case series seem to be in line with these earlier observations. AIH patients with anti-HEV-IgG antibodies did not show any significant differences in specific characteristic as compared to anti-HEV-negative AIH patients. In contrast to the previous studies, two cross-sectional control groups of patients another hepatic disease or with another autoimmune respectively have been studied. Of note, even though RA patients were older than patients with AIH, the anti-HEV prevalence was more than two-fold higher in AIH as compared to RA patients. Moreover, the seroprevalence rate of HBV- or HCV-infected patients was the similar to healthy individuals and more than three times lower than in AIH patients. Of note, the higher anti-HEV seroprevalence rate of AIH patients compared to patients with chronic viral hepatitis was confirmed by a second independent sero-assay, the Wantai assay, which has been suggested to be more sensitive and to detect much higher frequencies of anti-HEV [16], [17]. Even though the “background” seroprevalence rate in chronic viral hepatitis patients was indeed almost 10-fold higher using the Wantai-assay, AIH patients were again about 1.5 fold more likely to test anti-HEV positive. Still, 95% confidence intervals overlapped in this analysis and larger cohorts would therefore need to be studied to confirm or disprove our findings.

What could be possible explanations for an increased prevalence of anti-HEV antibodies in patients with autoimmune hepatitis? One possibility might be that previous subacute HEV infections could have triggered immune events leading to the manifestation of AIH. This hypothesis would be in line with several previous studies describing an association between viral infections and subsequent development of AIH (2, 4). Specific cross-reactive humoral or T cell epitopes cross-reacting with possible auto-antigens have been described for HCV infection [20]. If these are also present during HEV infections remains to be shown. Larger studies are necessary to confirm the association between AIH and HEV-seropositivity and to get more insights into the underlying pathophyisiology.

Second, patients with autoimmune hepatitis may have distinct risk factors for acquiring HEV infections. This could include more frequent blood transfusions and medical procedures as well more frequent contacts to animals representing a potential zoonotic reservoir for HEV infection. Of note, not only pigs [21] but also several other animal species such as cats [22], shellfish [23], deer [24] or rats [25] can be carriers of HEV. Third, cross-reactive non-HEV-specific antibodies could be present in patients with AIH. The specificity of most HEV ELISA tests is not 100% but conditions determining “false-positive” results are currently unknown. Fourth, acute hepatitis E may present with histological and biochemical features of AIH and thus acute disease may be misclassified as de novo onset of AIH if HEV infection has not been excluded [26].

Persistent HEV infections have been described in organ transplant recipients receiving higher doses of immunosuppression and HIV-infected individuals with particular low CD4 counts. Studying 208 AIH patients receiving low doses of steroids alone or in combination with azathioprine, we could not identify a single case of chronic hepatitis E. This is an important clinical information suggesting that the risk to develop chronic hepatitis E should not been overestimated in patients with lower levels of immunosuppression in a non-endemic area. This finding is also well in line with our experience in patients with HIV-infection [27]. However, HEV infections can occur and thus hepatitis E should be considered in the differential diagnosis when AIH patients seem to be non-responder to immunosuppressive treatment.

Control of viral infections is believed to be mediated by both humoral and cellular immune responses. Interestingly, we detected a strong and multispecific HEV-specific T cell responses in an AIH patient who had cleared acute HEV infection previously while responses were much weaker when she was still HEV viremic. In addition, HEV-specific T cell responses were detected in three anti-HEV-positive AIH patients suggesting that these individuals had indeed contact with HEV and that unspecific cross-reactivity of the antibody assay seems to be unlikely. Future studies should investigate mechanisms potentially leading to the failure to mount an efficient adaptive cellular immune response against HEV. Overall, the findings presented in this paper are well in line with our experience in studying HEV-specific T cell responses in organ transplant recipients where both CD4+ and CD8+ T cell responses became detectable only after clearance of HEV RNA [15].

In conclusion, we show that patients with autoimmune hepatitis have a higher prevalence of anti HEV antibodies than control populations. The underlying cause for this observation remains to be determined. In addition, we did not find any evidence for an increased prevalence of chronic hepatitis E in German individuals with autoimmune hepatitis receiving standard immunosuppressive medications. However in single cases HEV infections may occur and thus hepatitis E needs to be considered in the differential diagnosis of non-response to immunosuppressive medications in AIH.
